# Lipozyme 435-Mediated Synthesis of Xylose Oleate in Methyl Ethyl Ketone

**DOI:** 10.3390/molecules26113317

**Published:** 2021-06-01

**Authors:** Maria Carolina Pereira Gonçalves, Jéssica Cristina Amaral, Roberto Fernandez-Lafuente, Ruy de Sousa Junior, Paulo Waldir Tardioli

**Affiliations:** 1 Postgraduate Program in Chemical Engineering, Laboratory of Enzyme Technologies (LabEnz), Department of Chemical Engineering, Federal University of São Carlos (UFSCar), Rodovia Washington Luís, km 235, São Carlos 13565-905, SP, Brazil; mariacarolinapgoncalves@gmail.com; 2 Postgraduate Program in Chemistry, Laboratory of Natural Products, Department of Chemistry, Federal University of São Carlos (UFSCar), Rodovia Washington Luís, km 235, São Carlos 13565-905, SP, Brazil; jessica.camaral@yahoo.com.br; 3 Departamento de Biocatálisis, ICP-CSIC, Campus UAM-CSIC, 28049 Madrid, Spain; 4 Center of Excellence in Bionanoscience Research, Member of the External Scientific Advisory Board, King Abdulaziz University, Jeddah 21589, Saudi Arabia; 5 Postgraduate Program in Chemical Engineering, Laboratory for Development and Automation of Bioprocesses (LaDABio), Department of Chemical Engineering, Federal University of São Carlos (UFSCar), Rodovia Washington Luís, km 235, São Carlos 13565-905, SP, Brazil; ruy@ufscar.br

**Keywords:** sugar fatty acid esters, xylose, oleic acid, Lipozyme 435, methyl ethyl ketone

## Abstract

In this paper, we have performed the Lipozyme 435-catalyzed synthesis of xylose oleate in methyl ethyl ketone (MEK) from xylose and oleic acid. The effects of substrates’ molar ratios, reaction temperature, reaction time on esterification rates, and Lipozyme 435 reuse were studied. Results showed that an excess of oleic acid (xylose: oleic acid molar ratio of 1:5) significantly favored the reaction, yielding 98% of xylose conversion and 31% oleic acid conversion after 24 h-reaction (mainly to xylose mono- and dioleate, as confirmed by mass spectrometry). The highest Lipozyme 435 activities occurred between 55 and 70 °C. The predicted Ping Pong Bi Bi kinetic model fitted very well to the experimental data and there was no evidence of inhibitions in the range assessed. The reaction product was purified and presented an emulsion capacity close to that of a commercial sugar ester detergent. Finally, the repeated use of Lipozyme 435 showed a reduction in the reaction yields (by 48 and 19% in the xylose and oleic acid conversions, respectively), after ten 12 h-cycles.

## 1. Introduction

Sugar fatty acid esters (SFAEs) are non-ionic, low toxic, and biodegradable surfactants widely used for food processing, personal care, protein extraction, and as antimicrobial and insecticidal agents [1,2,3,4]. Particularly, xylose fatty esters have been used to improve skin hydration in the cosmetic and pharmaceutical sectors [1]. Xylose is a monosaccharide that can be used as a renewable, low-cost, and abundant raw material for SFAEs production. It can be obtained from the hydrolysis of hemicellulose, one of the main components of the lignocellulosic biomass [5,6]. However, the use of xylose in the synthesis of SFAEs has been sparsely reported in the literature [1,5,6,7,8,9,10,11,12], making room for the development of research in this area. Considering this scenario, xylose may represent a feasible substrate in the production of SFAEs, since, after the hydrolysis of lignocellulosic biomasses, the hemicellulose fraction is generally underused [12].

The synthesis of xylose fatty esters may occur through either an esterification or a transesterification reaction in the presence of chemical catalysts or enzymes. Xylose acts as the acceptor for the acyl group, while fatty acids/fatty acid esters perform as donors (or activated acyl donors) of this acyl group [13,14]. In the chemical route, the reaction is carried out at high temperatures with an alkaline or metallic catalyst. This pathway consumes a large amount of energy, presents low selectivity and the purification of the esters formed is complex [6,15], because of the toxic by-products produced [16]. Conversely, the production of these surfactants through an enzymatic route requires milder operating conditions, facilitates the separation of products, achieves high yields, and presents high specificity and selectivity [5,11,16,17]. Nonetheless, nowadays this route is not the most feasible option for large-scale production. It is still necessary to reduce the cost of biocatalysts and eliminate expensive processing steps.

Lipases (triacylglycerol hydrolases, E.C. 3.1.1.3) are the most used enzymes in the enzymatic synthesis of SFAEs. Several lipases have been evaluated for this purpose, among them lipases from *Candida antarctica*, *Candida rugosa*, *Candida cylindracea*, *Rhizomucor miehei*, *Bacillus subtilis*, *Bacillus licheniformis*, and the porcine pancreatic lipase [7,8,16,18]. In particular, the species *Candida antarctica* produces two different lipases: A and B. Fraction B (CALB) is probably the most widely used hydrolase in the field of biocatalysis, conducting reactions with high activities and yields under mild conditions [19]. CALB is a highly selective and efficient catalyst, applied in numerous organic reactions, with emphasis on the esterification in solvent-free systems and organic solvents [1,10,20,21].

The use of such enzymes as catalysts in the free form presents some issues related to the instability of their structures and the difficulty of recovery and reuse at the end of the process [22]. To minimize these drawbacks, CALB immobilization was addressed in about 95% of the publications that encompass the synthesis of SFAEs (in the time span from 2010 to 2019) [23]. The interfacial adsorption of lipases on hydrophobic supports [24], covalent bonds, and ionic interactions are among the immobilization techniques reported in this period [25]. These methods included the use of different supports, such as chitosan, celite, silica magnetic microparticles, octyl silica [26,27,28,29], and microemulsion-based organogels (MBGs) [30,31].

Specifically, the use of the commercial CALB physically adsorbed on a macroporous resin (Novozym 435 and Lipozyme 435) was reported in about 80% of the publications on the synthesis of sugar esters over the last decade [23]. Novozym 435 and Lipozyme 435 are CALB immobilized on Lewatit VP OC 1600, a poly methacrylic acid cross-linked with divinylbenzene. The described loading of CALB onto this support ranges between 8.5 and 20% (*w*/*w*) [32]. CALB immobilized on acrylic resin has outstanding activity and stability in hydrophobic organic media, besides producing high yields of carbohydrate esters when used in these reactions [5,6,28,33,34,35,36]. However, due to enzyme displacement or leaching from the support, multiple reuse cycles of Novozym 435 are often unfeasible [32,37]. Even so, its use is a good first approach to analyze the feasibility of the CALB use in a specific process.

The solvent used in the CALB catalyzed synthesis of SFAEs is another variable that should be carefully evaluated. It must reconcile sugar solubility and low polarity to reduce the damage on the enzyme structure and does not present reactive hydroxyl groups, which could be substrates of the enzyme and reduce the product yield and/or generate by-products. In general, tertiary alcohols meet these requirements [38,39,40]. Nonetheless, many of them are not permitted for the manufacture of food additives and should be replaced by biocompatible solvents. Methyl ethyl ketone (MEK) is an example of these solvents useful for food manipulation and has given promising results in the synthesis of sugar esters, even with low to moderate solubilization of sugars [1,41].

In this new research effort, we evaluated the performance of the benchmark immobilized CALB (Lipozyme 435) on the synthesis of xylose oleate in MEK to obtain the most suitable esterification conditions. The esterification reaction is a controlled process where the yields are determined by the system’s thermodynamics. The catalysts only determine the reaction feasibility, i.e., if the enzyme is inhibited or inactivated, the thermodynamic yield will be not reached [42,43]. Water is a by-product of this reaction that can be eliminated by diverse techniques to improve the yields (evaporation, molecular sieves, etc.) [44,45]. However, if the biocatalyst activity is high enough, the accumulation of water in the support particles may become a problem. If a water phase is formed, it can drive to the inhibition or inactivation of the enzyme [46,47]. This issue has been solved by some authors using very hydrophobic supports or ultrasounds [40,48,49,50,51,52,53,54]. Additionally, we assessed the kinetic parameters of the reaction, the biocatalyst reuse, the purification of the sugar esters synthesized, and their emulsion capacity for further industrial application with a commercial surfactant as a comparison. The successful formation of esters was evaluated by mass spectroscopy.

## 2. Results and Discussion

### 2.1. Enzymatic Synthesis of Xylose Oleate

#### 2.1.1. Effect of the Xylose: Oleic Acid Molar Ratio on the Process Performance

**Figure 1:**
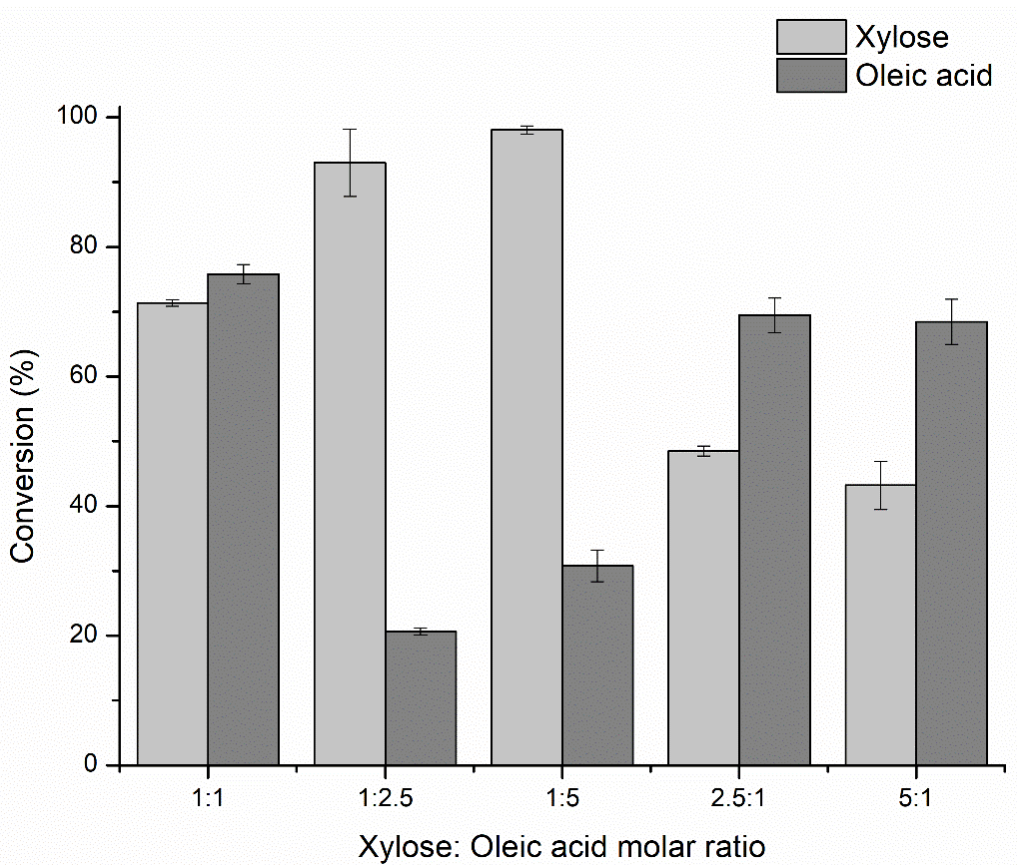
Optimization of the xylose: oleic acid molar ratio in the synthesis of xylose oleate in methyl ethyl ketone. Reaction conditions: 60 °C, 24 h-reaction, 200 rpm, 7 mM of xylose, 0.5% *w*/*v* of Lipozyme 435, with molecular sieves.

After evaluating the xylose solubility in MEK (7.16 ± 0.35 mM), we assessed the xylose/oleic acid molar ratios in the esterification reaction to optimize the production of xylose oleate. From the results shown in Figure 1, an excess of oleic acid (1:5 molar ratio), that turns the hydroxyl and acid molar ratio into 1:1.25, had a positive effect on the reaction, leading to a consumption of 98% xylose and 31% oleic acid. This suggested that xylose was modified with one or two molecules of oleic acid, as the ratio is 1.25 molecules of oleic acid per hydroxyl group of each xylose molecule. Very likely the lipase selectivity prevented the incorporation of more fatty acid molecules. Pappalardo et al. [36] reported that in the esterification of palmitic acid with L-(+)-arabinose (1:1 molar ratio) the Novozym 435 preferentially catalyzed the acylation of the primary hydroxyl group at the C-5 position of the carbohydrate because the secondary hydroxyl groups are less accessible to the active site of the enzyme. In fact, when we used the xylose: oleic acid molar ratio of 1:1, both xylose and oleic acid conversions were very close (around 70 and 75%, respectively, Figure 1), indicating that almost only one hydroxyl group of the xylose were substituted. 

However, the use of an excess of fatty acid enhances the substrate availability for the enzyme and facilitates the acylation, also shifting the equilibrium in the direction of the synthesis, thus, leading to higher xylose conversions [55]. Some studies addressing the lipase-catalyzed synthesis of xylose fatty esters also used an excess of fatty acid to sugar, as shown in Table 1.

Conversely, an excess of sugar had a negative effect on the reaction performance, with lower yields even with respect to the acid. These lower yields after 24 h should be related to a kinetic problem, since thermodynamically the yields regarding the minority substrate should increase. It seems that oleic acid has some positive effect on the reaction kinetics, and its reduction produces some negative effects. Perhaps, the acylation of the enzyme by the acid is the limiting step of the reaction and when decreasing the concentration of the acid, the effect on the reaction rate is rather significant (in fact, as determined in Section 2.1.3, the Km for this compound is high—see below). Moreover, an excess of oleic acid (a mild detergent) may increase the solubility of the xylose in the solvent or prevent its adsorption on some surfaces. Finally, it can produce some positive changes in the enzyme conformation. Although it has been shown that an excess of oleic acid prevents the xylose adsorption to the molecular sieves (as described below), the other effects may also cause a decrease in the yields when the oleic acid concentration decreases.

When the molar ratio of 1:5 was used, if the reaction produced only monoesters, the maximum theoretical oleic acid conversion should be ~20%, regarding the xylose conversion of 98%. Nonetheless, the reaction achieved experimentally around 31% oleic acid conversion, suggesting that not only monoesters were produced (in fact, mass spectrometry later confirmed the presence of mono- and diesters, besides the presence of some minority of triesters—Section 2.2, see below). The formation of this mixture of esters may be due, among other factors, to the solvent used in the reaction, as reported by Li et al. [41], who described significant differences in the diester/monoester molar ratios after the direct esterification of fructose with lauric acid catalyzed by Novozym 435 with 2-methyl-2-butanol (2M2B) and MEK as solvents. In that paper, when using MEK, the diester/monoester molar ratio was ~3:1, while when using 2M2B, it was 1:1. Furthermore, when the authors directly employed monoester and the fatty acid in the synthesis of diesters, the maximum initial rate of diester synthesis in MEK was 2.2-fold greater than the rate in 2M2B, indicating that CALB conformation in MEK preferred the monoesters as substrate more than in 2M2B. Therefore, the use of MEK as a solvent may partially explain the formation of diesters pointed out here. Although the CALB is reported to produce preferentially sugar monoesters [9,36], intramolecular acyl migrations can occur, mainly during long time processing, thus allowing to produce heterogeneous mixtures of esters with different degrees of acylation, a typical outcome observed in chemical processing of sugar esters, requiring laborious and costly protection and deprotection of hydroxyl groups [16,57,58,59].

To synthesize only monoesters using the molar ratios of 2.5:1 and 5:1, the highest xylose conversions should be theoretically 28% for an oleic acid conversion of ~70% and 14% for an oleic acid conversion of 68.5%, respectively. However, experimentally higher xylose conversions were reached (48.5% for the 2.5:1 molar ratio and 43.2% for the 5:1 molar ratio as shown in Figure 1), which are not plausible considering that the acid was used as a limiting reagent in this set of experiments. The logical explanation for this is that xylose may be somehow decomposing, precipitating, or reacting, and when the yields are calculated, they are overestimated. The presence of the xylose degradation product [60] (furfural, Section 3.6.3) was not observed.

After discarding the modification of xylose to furfural, we checked if this sugar can be adsorbed in some of the material employed in the reaction. Thus, we conducted some assays to verify if the xylose depletion only occurs for its consumption during the esterification reaction, and not by long time precipitation in the presence of the reaction product, which could affect the calculation of the conversions based on remaining unmodified xylose. We found that the final precipitate not dissolved in water (from the samples to be quantified by HPLC analysis—Section 3.6.1) did not contain xylose.

We also washed the biocatalyst before and after the reaction (24 h) with water and a high ionic strength buffer and performed the subsequent xylose quantification, to check if xylose could be adsorbed in the support. The same procedure was conducted with the molecular sieves (MS) used in the reaction to capture the released water [61,62,63]. Results demonstrated that the sugar was not adsorbed on Lipozyme 435, while 11.23 ± 2.20% of the xylose was adsorbed on MS at the end of the reaction when an excess of xylose was used. The synthesis of xylose oleate catalyzed by the soluble CALB (Lipozyme CALB L) was also conducted under the conditions aforementioned. The xylose adsorption percentage on MS was 14.32 ± 5.37%. We performed this same approach within only 15 min of reaction catalyzed by Lipozyme 435 and verified that 10.87 ± 0.82% of the xylose was adsorbed on MS (again only when using an excess of xylose). This means that, in this case (i.e., excess of sugar), after only 15 min of reaction, MS surface retained a significant percentage of our hydrophilic by-product (residual xylose). This, in our assay, was confused with a conversion of this reagent. When using an excess of oleic acid (1:2.5 and 1:5), the xylose was not adsorbed on MS at all, not being longer a problem.

Just for academic purposes, we used the mean of those results (11.05 ± 0.25%) as a correction factor for the observed xylose conversions using an excess of xylose (molar ratios of 2.5:1 and 5:1). Figure 1 shows the xylose conversions already corrected. With the molar ratios with an excess of fatty acid this correction was not necessary and so, the conversions remained the same. Though, the xylose conversions are still higher than those expected from the oleic acid conversion when the fatty acid was the minority reagent, suggesting that some other factor may be relevantly affecting this reaction when using low oleic acid (as discussed above) and must be deeper investigated in further studies. Nevertheless, we intend to have a full conversion of xylose to esters, therefore conditions using a molar excess of sugar were not interesting for our goal. That way, we have continued with the research using an excess of oleic acid.

Some studies already evaluated the use of molecular sieves in the enzyme-catalyzed synthesis of xylose fatty esters [6,12,27,56]. Among those authors, Tsukamoto et al. [56] found higher xylose conversions in the presence of MS when compared to the conversions in the absence of them. Nonetheless, they did not assess whether the xylose was adsorbed on the MS surface, which could mask the results. In view of our findings, we selected the xylose: oleic acid molar ratio of 1:5 for subsequent assays of xylose acylation.

#### 2.1.2. Effect of the Temperature on the Lipozyme 435 Activity Profile

**Figure 2:**
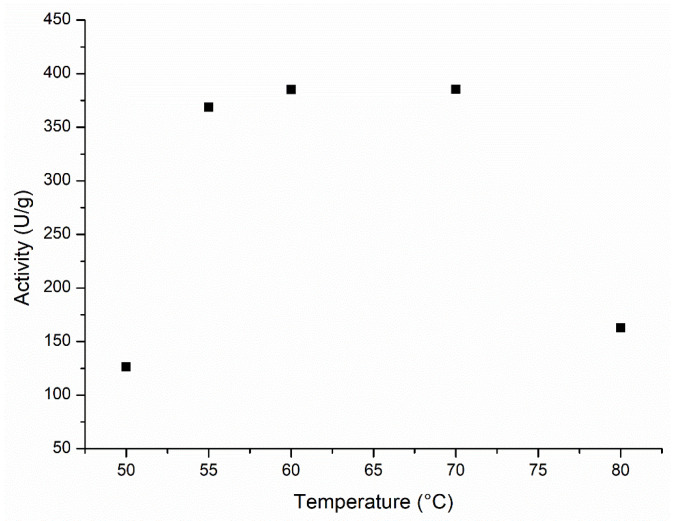
Activity profile as a function of the temperature for Lipozyme 435. Reaction conditions: xylose: oleic acid molar ratio of 1:5, 200 rpm, enzyme load of 0.16; 0.23 and 0.33% *w/v*, with molecular sieves, in methyl ethyl ketone.

Figure 2 shows the Lipozyme 435 activity profile in the esterification reaction for the temperature range from 50 to 80 °C. The highest activities (expressed as a function of the xylose consumption) occurred between 55 and 70 °C. A temperature higher than 70 °C greatly reduced the enzyme activity (>55%, at 80 °C) and therefore the substrate conversion.

Temperature influences both the reaction rate and the inactivation rate of the enzyme. At 50 °C the enzyme activity is about 30% of the maximum activity. Considering that there is no significant enzyme inactivation during the initial rate assays, it shows that the reaction is favored from 60 to 70 °C. The reduction in the enzyme activity observed for temperatures above 70 °C is very likely due to the enzyme thermal inactivation [64]. Ljunger, Adlercreutz, and Mattiasson [65], Lortie [66], Gbekeloluwa, Oguntimein, and Schmid [67], and Schlotterbeck et al. [68] also reported a similar behavior with the same biocatalyst in other esterification reactions. In our study, a temperature of 60 °C was chosen to perform the subsequent assays.

#### 2.1.3. Esterification Rates for Lipozyme 435

**Scheme 1:**
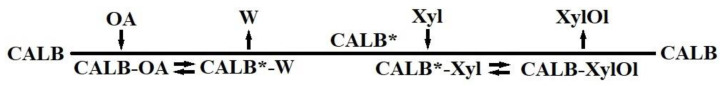
Ping-Pong Bi-Bi model of the reaction mechanism. OA is oleic acid, W is water, Xyl is xylose, and XylOl is xylose oleate.

The effects of a wide range of concentrations of both substrates (xylose and oleic acid) on the reaction rates were studied (see the reaction conditions in Section 3.2.4). The Ping-Pong Bi-Bi model was selected after a careful analysis of the literature to describe this reaction mechanism, as shown in Scheme 1. According to this mechanism, oleic acid initially binds to the CALB (Lipozyme 435) to form the non-covalent CALB-oleic acid (CALB-OA) that is subsequently transformed to an acyl-CALB intermediate, releasing the first product, water. Then, the acyl-CALB intermediate (CALB*) binds xylose (Xyl), forming the CALB-xylose oleate (CALB-XylOl), which then yields the ester product, xylose oleate (XylOl), and the free CALB (Lipozyme 435).

The rate equation for the Ping-Pong Bi-Bi mechanism is as follows:

(1) $$v=\frac{v_{máx}\cdot \left[OA\right]\cdot \left[Xyl\right]}{K_{m}{}_{\left(OA\right)}\cdot \;\left[Xyl\right]+K_{m}{}_{\left(Xyl\right)}\cdot \left[OA\right]+\left[OA\right]\cdot \left[Xyl\right]}$$

where v is the initial reaction rate, [OA] and [Xyl] are the respective concentrations of oleic acid and xylose, $v_{máx}$ is the maximum velocity or limiting rate, $K_{m}{}_{\left(OA\right)}$ and $K_{m}{}_{\left(Xyl\right)}$ are the respective Michaelis constants for oleic acid and xylose.

Kinetic constants of initial rate assays were estimated by Lineweaver-Burk plots generated by a linear regression method (OriginPro 8.5 Software) (Figures S1 and S2, Table S1—Supplementary Materials) and used as “initial guesses” in the fitting of the Ping-Pong Bi-Bi model to experimental data of a long-term assay (carried out in Force 2.0 Module). Table 2 shows the kinetic parameters for the model (Equation (1)) fitted to the long-term experimental data. They were of the same order of magnitude as those found by Zaidan et al. [69], who synthesized lactose fatty esters from capric acid in the presence of free lipase and lipase immobilized on a new support of mica clay. Moreover, it can be expected from the K_m_ values that commercial immobilized CALB (Lipozyme 435) has a poor affinity by both xylose and oleic acid, as the values are over 1 M for oleic acid and over 0.75 M for xylose. 

Li et al. [70] also found high Km values for lauric acid (11.6 M) and fructose (6.5 M) in the esterification catalyzed by Novozym 435 in 2M2B (50 °C, sugar/acid molar ratio of 1:1), using the Ping Pong Bi Bi model without inhibition. 

**Figure 3:**
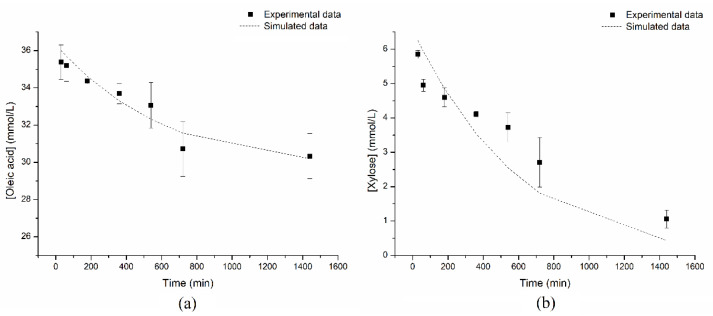
Plots of the experimental and simulated data of oleic acid (**a**) and xylose (**b**) consump-tion in the synthesis of xylose oleate in MEK catalyzed by Lipozyme 435. Reaction conditions (experimental data): 24 h-reaction; 60 °C; 2000 rpm; enzyme load of 1.0% (*w*/*v*); xylose: oleic acid molar ratio of 1:5; with molecular sieves.

Dang, Obiri, and Hayes [71] reported Km values of 0.4 M for both oleic acid and fructose in T-but, anew employing the non-inhibited Ping Pong Bi Bi model. The esterification was conducted at 65 °C, at a sugar/acid molar ratio of 1:1, and catalyzed by Lipozyme IM, a commercial lipase from *Rhizomucor miehei* immobilized on microporous anionic resin beads. The oleic acid and xylose consumption profiles of experimental and simulated data (from the kinetic constants calculated in this paper) are in Figure 3. Results indicated that the model fitted very well to the experimental data.

#### 2.1.4. Conversion Profiles in the Synthesis of Xylose Oleate

**Figure 4:**
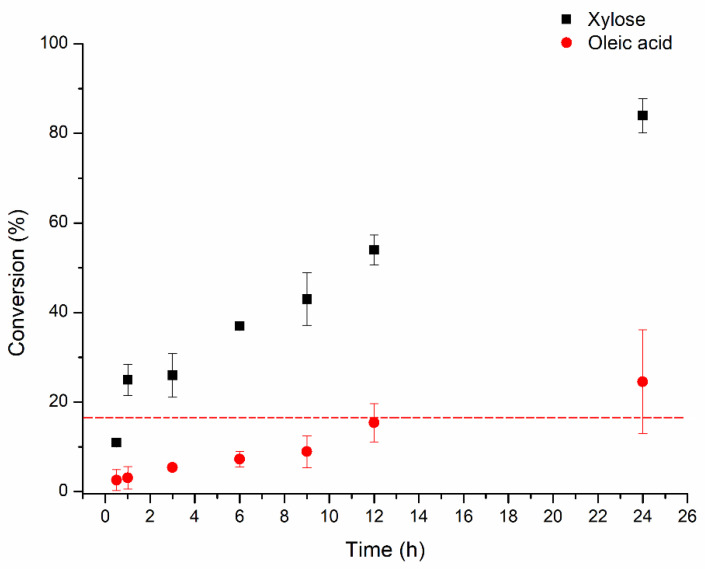
Xylose and oleic acid conversion profiles in the synthesis of xylose oleate catalyzed by Lipozyme 435 in a vortex flow reactor. The dotted line represents the expected maximum conversion of oleic acid regarding the modification of only one hydroxyl group of the xylose. Reaction conditions: 60 °C; 2000 rpm; enzyme load of 1.0% (*w*/*v*); xylose: oleic acid molar ratio of 1:5; with molecular sieves.

Xylose and oleic acid conversion profiles during the esterification reaction are presented in Figure 4. Here, we used an excess of fatty acid to xylose, since we already demonstrated that this condition provided higher conversions in the Lipozyme 435 catalyzed esterification reaction. Almost all xylose was consumed after 24 h of reaction (84%) and 24.6% of the oleic acid was depleted after the same period, anew suggesting that more than one hydroxyl group per xylose molecule was modified by the acyl moiety of the oleic acid. De Lima et al. [27] also reported a high xylose conversion (~65%) in the synthesis of xylose oleate catalyzed by immobilized CALB (CALB IM—T2-350) in tert-butyl alcohol (55 °C, xylose: oleic acid molar ratio of 1:5, 24 h). Vescovi et al. [12] achieved a xylose conversion around 70% (60 °C, 24 h, 1:5 molar ratio xylose/oleic acid in tert-butyl alcohol) in the synthesis of xylose oleate catalyzed by porcine pancreatic lipase immobilized on octyl-silica. Abdulmalek et al. [5] documented capric acid conversions ranging from 50 to 64% (depending on the solvent system, which could be: acetone, acetone:DMSO, T-but:DMSO, hexane:DMSO) in the synthesis of xylose caproate catalyzed by Novozym 435 at 60 °C after 24 h-reaction (xylose: capric acid molar ratio of 1:4). 

Bidjou-Haiour and Klai [1] achieved ~50% lauric acid conversion at 60 °C after 24 h using MEK as a cosolvent in the synthesis of xylose laurate catalyzed by Novozym 435 (xylose: lauric acid molar ratio of 1:1), so the abovementioned results emphasize the importance of the conversions achieved in this study through the use of the Lipozyme 435 biocatalyst in the synthesis of xylose fatty acid esters.

#### 2.1.5. Lipozyme 435 Operational Stability

**Figure 5:**
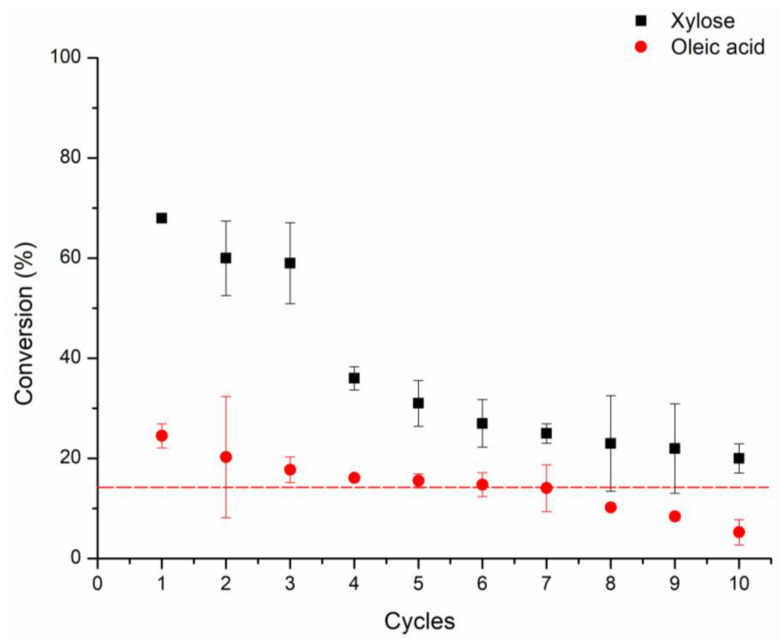
Lipozyme 435 operational stability. Reaction conditions of each batch: 60 °C; 2000 rpm; enzyme load of 10% (*w*/*v*); xylose: oleic acid molar ratio of 1:5; 12 h-cycles; with molecular sieves.

In industrial applications, recovery and reuse (recycling) of enzymes is often an essential cost-reduction measure. The effect of repeated use of Lipozyme 435 on the substrates conversions was investigated in successive reaction batches (Figure 5). Results demonstrated a decrease of 48 and 19% in the xylose and oleic acid conversions, respectively, after ten 12 h-cycles. Vescovi et al. [29] also reported a conversion decrease of 31% (in the synthesis of fructose oleate) after nine 6 h-batches with the Novozym 435. Ward et al. [72] described a drop of 55% in the conversions after eight 24 h reaction cycles. They synthesized a sugar ester from 1,2-o-isopropylidene-n-xylofuranose and arachidonic acid. Moreover, the authors estimated that 8–10% of the Novozym 435 activity was lost per cycle at the end of the reuse. In another study, the conversion of glucose esters from different N-fatty acyl glycines decreased 30% for the 5th 9 h cycle with Novozym 435 [73].

This decrease in the conversions over the reuse could be partially explained by the CALB desorption from the support while the product is engendered, as was reported by De Lima et al. [27], Neta et al. [28], and Vescovi et al. [29]. Indeed, we verified a reduction of ~50 ± 0.77% in the Lipozyme 435 hydrolytic activity (Section 3.5.1) after 12 h of incubation in the reaction medium of xylose oleate synthesis.

Since Lipozyme 435 presents the CALB non-covalently linked to the support (Lewatit VP OC 1600), this behavior could be attributed to some main aspects that may greatly facilitate the CALB release from the matrix: (1) a high temperature may weaken the enzyme-support interaction [74] (although this interaction is very strong, CALB has a very small lid) [75]; (2) high concentrations of substrates-products with surfactant properties (e.g., oleic acid and xylose oleate) could break those interactions and anew cause the lipase release to the medium [76].

Another fact that could decrease the operational stability of the CALB is the incomplete removal of the compounds adsorbed to its surface between each batch. For instance, water (a side-product of the esterification reaction) is adsorbed on molecular sieves to shift the equilibrium to the direction of the synthesis [45,63]. If this water formation is more rapid than the diffusion, and the support pores or matrix is more hydrophilic than the reaction medium, water can be accumulated inside the biocatalyst forming an aqueous phase [49]. This way, acids can also be accumulated in this environment, exposing the enzyme to low pH values and promoting enzyme inactivation by these drastic conditions [77]. Furthermore, water accumulation could make the thermodynamics of the process unfavorable in the enzyme proximity, reducing the enzyme activity [37].

Additionally, a minor reason for these reductions in the conversions could be the known mechanical fragility of Lewatit under stirring [78,79]. This may be an important negative feature when considering the use of Lipozyme 435 in mechanically stirred batch reactors. Nonetheless, this issue was minimized with the use of a stirred reactor configuration less aggressive with the mechanical structure of the beads (a vortex flow reactor) [80]. Further, the support solubility in MEK could represent another cause of the low operational stability of Lipozyme 435 in the synthesis of SFAEs that cannot be discarded [74].

These drawbacks have been considered as the main problems for the industrial implementation of Lipozyme 435 [81]. Thus, in accordance with other authors, we demonstrated the limited repeatability and stability of Lipozyme 435, which are crucial factors for cost reduction in large-scale sugar esters production. This could be solved using a lower temperature, which will require longer reaction times. The final decision will depend on the weight that the different parameters may be given in a specific factory (necessity of the use of a fresh enzyme or enlarge the reaction cycles). Another alternative may be to improve the biocatalyst stability by using a better support or by modifying the Lipozyme 435 surface [82,83,84].

### 2.2. Purification and Characterization of Xylose Oleate

The esterification reaction here described results in a crude sugar ester reaction product containing the sugar ester (xylose oleate), the biocatalyst (Lipozyme 435), the anhydrous solvent (MEK), as well as unreacted sugar (xylose), and unreacted fatty acid (oleic acid). So, a method for purifying and separating the sugar ester from the crude reaction product is required.

Samples of the crude sugar ester reaction product from the esterification of xylose with oleic acid were purified according to an adaptation of the methodology described by Wagner et al. [85] (Section 3.3). Most of the unreacted xylose and fatty acid molecules were removed after this process (97.01 and ≥89.64%, respectively).

To confirm the successful enzymatic formation of xylose oleate, the purified samples were then characterized by mass spectrometry (see Figures S3–S5 in the Supplementary Materials). Mass spectra of the [M-H]^−^ ion with *m*/*z* = 413.2 and 677.5 revealed that Lipozyme 435 mainly produced xylose mono- and dioleate (xylose: oleic acid molar ratio of 1:5). To a lesser extent, we also detected the presence of xylose trioleate (*m*/*z* = 941.7). Therefore, the possible formation of a mixture of esters mentioned above (Section 2.1.1) was confirmed here by mass spectrometry. Although immobilized CALB (Novozym 435) has been reported to be selective to modify only one hydroxyl group at the C5 primary position [36], it has also been described that the solvent can change this behavior, with MEK preferentially leading to the formation of diester/monoester at an approximate molar ratio of 3:1 [41].

### 2.3. Emulsion Capacity Assays

SFAEs are nonionic surfactants that exhibit emulsifying, stabilizing, and detergency effects, thus finding applications in the food, cosmetic, detergent, and pharmaceutical industry [8]. The emulsion capacity (EC) of the sugar ester synthesized and purified in this study (xylose oleate in MEK using a xylose: oleic acid molar ratio of 1:5) was of the same order of magnitude of a commercial sugar ester (sucrose monolaurate), 6.25 and 10%, respectively. This may enable the future application of those esters as industrial surfactants, along with other indicators to be assessed. The EC is highly correlated with the medium hydrophobicity [86]. Sugar esters are molecules bearing a hydrophilic and a hydrophobic moiety. The hydrophobic one consists of a fatty acid, whereas the hydrophilic one consists of a sugar molecule [86]. As the proportion of hydrophobic moieties of the molecule increases, the emulsion capacity is significantly reduced [87]. Thus, as sugar esters afford mixtures of compounds differing in their degree of esterification and/or the position of acylation, our product may contain more than one fatty acid residue (as confirmed by mass spectrometry) and, consequently, its hydrophobicity is enhanced. This means that esterification reactions that preferentially produce carbohydrate monoesters (which is the case of the commercial surfactant evaluated in this study) instead of a mixture of esters tend to present higher emulsion capacities and so, emulsion stabilities, compared to the last ones. Thus, the favored formation of sugar monoesters may represent an attractive approach for their commercial application [8,88].

## 3. Materials and Methods

### 3.1. Materials

*Candida antarctica* lipase B immobilized on Lewatit VP OC 1600 (Lipozyme 435) was donated by Novozymes Latin America (Araucária, Brazil). Tributyrin, butyric acid, and n-butanol were purchased from Sigma-Aldrich Co. (St. Louis, MO, USA). Xylose, heptane, and oleic acid were obtained from Synth (São Paulo, Brazil). MEK was purchased from Neon (São Carlos, Brazil). Molecular sieves (3 Å) were obtained from JT Baker (New Jersey, NJ, USA). All other reagents and solvents were of analytical or HPLC/GC grade and were used without any previous treatment.

### 3.2. Enzymatic Synthesis of Xylose Oleate

The synthesis of xylose oleate was conducted through the esterification reaction between oleic acid (C18:1) and commercial xylose (as a model compound), using MEK as solvent. The biocatalyst used in this reaction (Lipozyme 435) contains 1432 U of esterification/g of the enzyme. One unit of esterification activity (U) was defined as the initial rate of butyl butyrate synthesis (mmol/L/min) under the conditions described in Section 3.5.2. All experiments were performed in duplicate. Results were expressed as mean ± mean standard deviation (ϭ).

#### 3.2.1. Xylose Solubility in MEK

The xylose solubility was evaluated in the chosen organic solvent. To this goal, we added 50, 25, 10, and 5 mmol/L of xylose in MEK in closed tubes. After 24 h under continuous stirring (200 rpm) in a Marconi shaker (MA 430/1) (Marconi^®^, Piracicaba, Brazil) at 60 °C, the solutions or suspensions were centrifuged at 10,000 rpm (12,857× *g*) for 2 min (35 °C). The supernatant was then prepared for the quantification of solubilized xylose by liquid chromatography (Section 3.6.1—see below).

#### 3.2.2. Determination of the Effect of Xylose: Oleic Acid Molar Ratio on the Reaction Performance

The effect of xylose: oleic acid molar ratio in the synthesis of xylose oleate was studied under the following conditions: molar ratios of 1:1, 1:2.5, 1:5, 2.5:1, and 5:1, with the xylose concentration fixed at 7 mmol/L; 0.22 g of molecular sieves/g of water produced; 0.5% *w*/*v* of Lipozyme 435; temperature of 60 °C for 24 h under stirring (200 rpm) in a Marconi shaker. Subsequently, the biocatalyst was separated by centrifugation (10,000 rpm, 2 min, 35 °C) and 1 mL of the supernatant was dried at 70 °C for 24 h. After evaporating the solvent, samples were prepared for the quantification of unconverted xylose and oleic acid by liquid and gas chromatography, see Section 3.6.1 and Section 3.6.2, respectively.

#### 3.2.3. Effect of the Temperature on the Lipozyme 435 Activity Profile

The esterification reaction was conducted at different temperatures (50, 55, 60, 70, 80 °C) with 0.17, 0.22, and 0.33% *w*/*v* of Lipozyme 435 at the following conditions: 200 rpm, xylose: oleic acid molar ratio of 1:5, 0.22 g of molecular sieves/g of water produced. Samples were collected over 5, 10, 15, 20, and 30 min of reaction and the initial xylose consumption rates were determined according to the methodology described in Section 3.6.1 (see below). Enzymatic activities were then obtained in U/g, where 1 Unit represents the consumption of 1 µmol of xylose/minute/gram of Lipozyme 435.

#### 3.2.4. Determination of Esterification Rates for Lipozyme 435

##### Determination of Initial Rates

Esterification reactions were conducted at 60 °C under continuous stirring (200 rpm) for 15 min, with 0.23% *w*/*v* of Lipozyme 435 and 0.22 g of molecular sieves/g of water produced. In one set of experiments, the xylose concentration was varied from 0.28 to 7 mmol/L with a fixed quantity of oleic acid (1.4 mmol/L), while in another set of experiments, the amount of oleic acid was varied from 1.4 to 106 mmol/L with a fixed quantity of xylose (7 mmol/L). The biocatalyst was separated by centrifugation at 10,000 rpm for 2 min (35 °C) and the supernatant was then prepared for the quantification of unconverted xylose as described in Section 3.6.1 (see below).

##### Determination of Long-Term Rates

The following conditions were used to conduct esterification reactions in a vortex flow reactor (80 mL): 60 °C; 2000 rpm; 1% *w*/*v* of Lipozyme 435; and xylose: oleic acid molar ratio of 1:5. The reactor specifications were: radius ratio (η = R_in_/R_out_) of 0.24, aspect ratio (Г = L/d) of 6.72, and rotation of the inner cylinder (metallic rod coupled to an IKA overhead stirrer, IKA Werke GmbH & Co. KG, Breisgau, Germany) at 2000 rpm stirring. Tests were performed in the presence of molecular sieves (0.22 g/g of water produced). Samples were collected after 30 min, 1 h, 3, 6, 9, 12, and 24 h of reaction, and quantified according to the methodologies presented in Section 3.6.1 and Section 3.6.2 (see below).

#### 3.2.5. Determination of the Lipozyme 435 Operational Stability

To evaluate the operational stability of the biocatalyst, the Lipozyme 435 catalyzed synthesis of xylose oleate was performed in 10 successive 12 h-reaction batches. Reactions were conducted in a vortex flow reactor under the following conditions: 60 °C, continuous stirring (2000 rpm), xylose: oleic acid molar ratio of 1:5 (7 mmol/L of xylose), 0.22 g of molecular sieves/g of water produced, 10% *w*/*v* of Lipozyme 435. After each batch, the biocatalyst and molecular sieves were recovered by centrifugation (35 °C; 10,000 rpm; 2 min) and washed with MEK at room temperature in a commercial sieve, which retained all molecular sieves. Lipozyme 435 was anew washed with MEK and subjected to vacuum filtration to remove the remaining solvent and reused in a new synthesis. Xylose and oleic acid conversions were measured by liquid and gas chromatography, as described in Section 3.6.1 and Section 3.6.2 (see below), respectively.

### 3.3. Purification and Characterization of Xylose Oleate

The purification of the crude sugar ester reaction product followed a protocol adapted from Wagner et al. [85]. In a beaker, 2 mL of the esterification reaction solution were added to 2 mL of an ethanol-water solution (50:50, *v*/*v*), and magnetically stirred. After complete homogenization, the stirring was stopped, and the solution was left standing for 1 h at room temperature. The precipitate was collected by centrifugation at 10,000 rpm for 15 min and mixed with 2 mL of the ethanol-water solution at room temperature. The ethanol-water solution washed precipitate was collected by centrifugation as described above. This last step was repeated (a total of two washes with alcohol-water solution). The recovered ethanol-water solution washed precipitate was then mixed with 2 mL of MEK at room temperature. The MEK-washed precipitate was recovered by centrifugation as previously described. This step was repeated three times and the recovered organic solvent washed precipitate was dried in a vacuum oven.

The purified xylose oleate was then characterized by mass spectrometry using an electrospray ionization quadrupole time-of-flight MS/MS operated in the negative mode on a 6545 ESI-QTOF-MS instrument (Agilent, Santa Clara, CA, USA)) across the mass range of 50–1200 Da. Samples were dissolved in methanol and analyzed by FIA at a flow rate of 0.35 mL/min and a volume injection of 5.0 µL at 38 °C. The mobile phase consisted of H_2_O + 0.1% formic acid and MeCN + 0.1% formic acid 20:80 with an analysis time of 4.0 min. The ESI source conditions were as follows: capillary voltage 2500 V, nozzle voltage 500 V, gas temperature 350 °C, drying gas 13 L/min, nebulizer 35 psi, sheaf gas 320 °C in a flow of 10 L/min, fragmentor 120 V, skimmer 80 V, and collision energy voltage of 20 and 22 V. Data were analyzed with the use of the Qualitative Navigator B.08.000 software.

### 3.4. Emulsion Capacity Assays

Emulsion capacity (EC) assays were performed by adding 10 mg of the purified reaction product and 2 mL of kerosene to a test tube. The content of the tube was continuously homogenized for 2 min at room temperature (25 °C) and then left standing for 24 h. Tests were performed in duplicate and the standard deviations were less than 1%. After this period, the height of the emulsified region and the height of the total column were measured, and the ECs were calculated according to Equation (2) [89]. Sucrose monolaurate was used as a standard for these experiments:

(2) $$EC\;\left(\%\right)=\frac{H_{e}}{H_{t}}\cdot 100$$

where $H_{e}$: height of the emulsified region and $H_{t}$: height of the total column.

### 3.5. Standard Enzymatic Activities

#### 3.5.1. Hydrolysis Activity of Lipozyme 435

The protocol to measure the hydrolysis activity of Lipozyme 435 in tributyrin is an adaptation of the methodology described by Beisson et al. [90]. The method is based on the hydrolysis of a mixture of 1.5 mL of tributyrin, 6 mL of 100 mmol/L phosphate buffer, pH 7.3, and 16.5 mL of distilled water, at 37 °C for 5 min. The released butyric acid was titrated with 0.02 mol/L KOH solution in pH-Stat titrator (Titrando 907, Metrohm, Herisau, Switzerland). A unit of tributyrin hydrolysis activity is defined as the amount of enzyme that releases 1 μmol of butyric acid per minute under the conditions described above.

#### 3.5.2. Esterification Activity of Lipozyme 435

The esterification activity of Lipozyme 435 was determined through the esterification of butyric acid (0.1 mol/L) with butanol (0.1 mol/L) in heptane (10 mL) at 37 °C. The reaction was conducted in agitated closed flasks (250–300 rpm) and monitored by the consumption of butyric acid by titration with 0.02 mol/L KOH solution, using phenolphthalein as an indicator [13,29,91].

### 3.6. Chromatographic Analyses

The reliability of all chromatographic methods applied in this research was confirmed by samples of known concentrations with amounts close to those expected (≥90% accuracy).

#### 3.6.1. Xylose Determination

The final reaction medium was centrifuged, and 1 mL of the supernatant was collected. After evaporating the solvent, 1 mL of water was added to the samples and after homogenization, it was filtered using 0.22 µm syringe filters. The xylose concentration was measured using an HPLC Breeze equipped with a refractive index detector (RID) and a Sugar Pak-I column (300 × 6.5 mm × 10 μm) maintained at 80 °C. A solution of EDTA-Ca (50 mg/L) was used as eluent, at 0.5 mL/min. The injection volume was 20 μL, with a run time of 20 min [12]. Similar conversions were identified by another method, which anew confirms the reliability of this protocol. The second one performs the washing and homogenization of the final reaction medium with distilled water and removes the aqueous phase for xylose quantification.

#### 3.6.2. Oleic Acid Determination

The reaction medium was centrifuged, and 1 mL of the supernatant was collected. The concentration of oleic acid in the samples was measured using an Agilent gas chromatograph 7890A equipped with a flame ionization detector (FID) and a Restek Rtx-wax column (30 m × 0.25 mm × 0.25 µm; Restek Corporation, Bellefonte, PA, USA) maintained at 200 °C for 1 min, 230 °C for 1 min (10 °C/min), and 250 °C for 5 min (5 °C/min). Helium was used as carrier gas at a flow rate of 1.8 mL/min, with a run time of 14 min [92].

#### 3.6.3. Furfural Determination

The presence of the xylose degradation product (furfural) was verified using an HPLC Breeze equipped with a UV–visible detector (274 nm) and a C-18 Ascentis Express column (10 cm × 46 mm × 2.7 μm, Supelco, Bellefonte, PA, USA) maintained at 40 °C. A solution of acetonitrile/water 1:8 (*v*/*v*) with 1% acetic acid was used as eluent, at 0.8 mL/min. The injection volume was 20 μL, with a run time of 15 min [93].

## 4. Conclusions

The benchmark immobilized CALB (Lipozyme 435) was proven to be efficient for xylose oleate synthesis in methyl ethyl ketone, yielding high substrates conversions. An excess of oleic acid markedly favored the reaction catalyzed by Lipozyme 435. Besides, when an excess of sugar was used, since the first 15 min of reaction, molecular sieves retained nearly 11% of our hydrophilic reagent (xylose), producing an apparent increase in the conversions. Thus, for this case, the use of different means of water removal from the product stream is required, such as salt hydrate pairs, saturated salt solutions, or pervaporation.

The predicted Ping Pong Bi Bi kinetic model presented an adequate fit to the experimental data (oleic acid and xylose consumption profiles) and the kinetic constants determined for this model revealed that Lipozyme 435 has a poor affinity for both substrates. The emulsion capacity of our purified product was close to that of a commercial product (also a sugar ester). Lipozyme 435, therefore, has considerable potential for use in the production of biosurfactants based on xylose and oleic acid. Nevertheless, its limited repeatability and stability turn the cost reduction in the large-scale xylose oleate production difficult. Moreover, the preferential formation of xylose monoesters may represent a better alternative for their commercial use, and, in this study, we identified the presence of a mixture of xylose mono-, di- and trioleate.

## Figures and Tables

**Table 1:** Components and conditions of the reaction system in the synthesis of xylose fatty esters and corresponding conversions.

Acyl Donor	MR *	Solvent	Conditions	Biocatalyst	Conversions *	References
Capric acid	1:4	DMSO; ACT	60 °C, 24 h	N435	64 (FAC)	[5]
Oleic/Lauric acids	1:5	T-but	46–55 °C, 48 h	CALB IM T2-350 CALB-SMMP-O CALB-SMMP-OG	XC: 52/54 (46 °C); 65 (55 °C) 50 (46 °C); 61/66 (55 °C) 57/50 (46 °C); 69/66 (55 °C)	[27]
Vinyl laurate	1:3	2M2B; HEX; THF	50 °C, 4 h	N435	74.8 (XC)	[6]
Butyric/caprylic/oleic acids	1:5	T-but, T-pent	60 °C, 24 h	PPL-OS	70 (XC)	[12]
Acrylic acid	1:4.2	T-but	55 °C, 48 h	CALB IM N	4.2 (XC)	[56]
Oleic acid	1:5	MEK	60 °C, 24 h	L435	98 (XC), 31 (FAC)	This study

MR *: Xylose: fatty acid molar ratio; Conversion *: %; T-but: Tert-Butyl alcohol; T-pent: Tert-Pentyl alcohol; 2M2B: 2-Methyl-2-butanol; THF: Tetrahydrofuran; DMSO: Dimethyl sulfoxide; ACT: Acetone; HEX: Hexane; N435: Novozym 435; L435: Lipozyme 435; CALB-SMMP-O: CALB immobilized on silica magnetic microparticles and functionalized with octyl groups; CALB-SMMP-O-G: CALB immobilized on silica magnetic microparticles and functionalized with octyl and aldehyde groups; PPL-OS: Porcine pancreatic lipase immobilized on octyl-silica; CALB IM N: CALB immobilized on a macroporous acrylic acid resin by Novo Nordisk A/S; FAC: Fatty acid consume; XC: Xylose consume.

**Table 2:** Kinetic constants of the Ping-Pong Bi-Bi model (Equation (1)) fitted to the enzymatic esterification data (fatty acid and sugar consumption).

Parameters	
V_max_ (mmol/L/min)	1.57
K_m(OA)_ (mM)	1120.40
K_m(Xyl)_ (mM)	754.16

Reaction conditions: 60 °C; 2000 rpm; 1.0% *w*/*v* of Lipozyme 435; xylose: oleic acid molar ratio of 1:5; with the addition of molecular sieves. Samples were collected after 30 min, 1 h, 3, 6, 9, 12, and 24 h of reaction.

## Supplementary Materials

The following are available online. Figure S1: Lineweaver-Burk plot for the initial reaction rates of xylose consumption for Lipozyme 435 at 1.4 mM of oleic acid, Figure S2: Lineweaver-Burk plot for the initial reaction rates of oleic acid consumption for Lipozyme 435 at 7 mM of xylose, Table S1: Kinetic constants of the esterification reaction catalyzed by Lipozyme 435, Figure S3: Mass spectrum of xylose monooleate with *m*/*z* = 413.2, Figure S4: Mass spectrum of xylose dioleate with *m*/*z* = 677.5, Figure S5: Mass spectrum of xylose trioleate with *m*/*z* = 941.7.

## Sample Availability

Samples of the compounds are not available from the authors.

## References

[1] Bidjou-Haiour C., Klai N. (2013). Lipase catalyzed synthesis of fatty acid xylose esters and their surfactant properties. Asian J. Chem..

[2] Chang P., Zhang Z., Tang S. (2018). Lipase-catalyzed synthesis of sugar ester in mixed biphasic system of ionic liquids and supercritical carbon dioxide. J. Chin. Chem. Soc..

[3] Ogawa S., Endo A., Kitahara N., Yamagishi T., Aoyagi S., Hara S. (2019). Factors determining the reaction temperature of the solvent-free enzymatic synthesis of trehalose esters. Carbohydr. Res..

[4] Shin D.W., Mai N.L., Bae S.W., Koo Y.M. (2019). Enhanced lipase-catalyzed synthesis of sugar fatty acid esters using supersaturated sugar solution in ionic liquids. Enzym. Microb. Technol..

[5] Abdulmalek E., Hamidon N.F., Abdul Rahman M.B. (2016). Optimization and characterization of lipase catalysed synthesis of xylose caproate ester in organic solvents. J. Mol. Catal. B Enzym..

[6] Méline T., Muzard M., Deleu M., Rakotoarivonina H., Plantier-Royon R., Rémond C. (2018). D-Xylose and L-arabinose laurate esters: Enzymatic synthesis, characterization and physico-chemical properties. Enzym. Microb. Technol..

[7] Chang S.W., Shaw J.F. (2009). Biocatalysis for the production of carbohydrate esters. New Biotechnol..

[8] Neta N.S., Teixeira J.A., Rodrigues L.R. (2015). Sugar ester surfactants: Enzymatic synthesis and applications in food industry. Crit. Rev. Food Sci. Nutr..

[9] Shi Y.G., Li J.R., Chu Y.H. (2011). Enzyme-catalyzed regioselective synthesis of sucrose-based esters. J. Chem. Technol. Biotechnol..

[10] Siebenhaller S., Hajek T., Muhle-Goll C., Himmelsbach M., Luy B., Kirschhöfer F., Brenner-Weiß G., Hahn T., Zibek S., Syldatk C. (2017). Beechwood carbohydrates for enzymatic synthesis of sustainable glycolipids. Bioresour. Bioprocess..

[11] Siebenhaller S., Kirchhoff J., Kirschhöfer F., Brenner-Weiß G., Muhle-Goll C., Luy B., Haitz F., Hahn T., Zibek S., Syldatk C. (2018). Integrated process for the enzymatic production of fatty acid sugar esters completely based on lignocellulosic substrates. Front. Chem..

[12] Vescovi V., dos Santos J.B.C., Tardioli P.W. (2017). Porcine pancreatic lipase hydrophobically adsorbed on octyl-silica: A robust biocatalyst for syntheses of xylose fatty acid esters. Biocatal. Biotransform..

[13] De Lima L.N., Mendes A.A., Fernandez-Lafuente R., Tardioli P.W., Camargo Giordano R.D.L. (2018). Performance of different immobilized lipases in the syntheses of short- and long-chain carboxylic acid esters by esterification reactions in organic media. Molecules.

[14] Liang M.Y., Chen Y., Banwell M.G., Wang Y., Lan P. (2018). Enzymatic preparation of a homologous series of long-chain 6- O -acylglucose esters and their evaluation as emulsifiers. J. Agric. Food Chem..

[15] Bouzaouit N., Bidjou-Haiour C. (2015). Optimization of lipase catalyzed synthesis of fatty acid xylose ester using statistical experimental designs. Pharma Chem..

[16] Gumel A.M., Annuar M.S.M., Heidelberg T., Chisti Y. (2011). Lipase mediated synthesis of sugar fatty acid esters. Process Biochem..

[17] Kapoor M., Gupta M.N. (2012). Lipase promiscuity and its biochemical applications. Process Biochem..

[18] Teng Y., Stewart S.G., Hai Y.W., Li X., Banwell M.G., Lan P. (2020). Sucrose fatty acid esters: Synthesis, emulsifying capacities, biological activities and structure-property profiles. Crit. Rev. Food Sci. Nutr..

[19] Kundys A., Białecka-Florjańczyk E., Fabiszewska A., Małajowicz J. (2018). Candida antarctica lipase B as catalyst for cyclic esters synthesis, their polymerization and degradation of aliphatic polyesters. J. Polym. Environ..

[20] Arcens D., Grau E., Grelier S., Cramail H., Peruch F. (2018). 6-O-glucose palmitate synthesis with lipase: Investigation of some key parameters. Mol. Catal..

[21] Ma Y.R., Banwell M.G., Yan R., Lan P. (2018). Comparative study of the emulsifying properties of a homologous series of long-chain 6′-O-acylmaltose esters. J. Agric. Food Chem..

[22] Gonçalves M.C.P., Kieckbusch T.G., Perna R.F., Fujimoto J.T., Morales S.A.V., Romanelli J. (2019). Trends on enzyme immobilization researches based on bibliometric analysis. Process Biochem..

[23] Gonçalves M.C.P., Romanelli J.P., Guimarães J.R., Vieira A.C., de Azevedo B.P., Tardioli P.W. (2021). Reviewing research on the synthesis of CALB-catalyzed sugar esters incorporating systematic mapping principles. Crit. Rev. Biotechnol..

[24] Rodrigues R.C., Virgen-Ortíz J.J., dos Santos J.C.S., Berenguer-Murcia Á., Alcantara A.R., Barbosa O., Ortiz C., Fernandez-Lafuente R. (2019). Immobilization of lipases on hydrophobic supports: Immobilization mechanism, advantages, problems, and solutions. Biotechnol. Adv..

[25] Adlercreutz P. (2013). Immobilisation and application of lipases in organic media. Chem. Soc. Rev..

[26] Chávez-Flores L.F., Beltran H.I., Arrieta-Baez D., Reyes-Duarte D. (2017). Regioselective synthesis of lactulose esters by *Candida antarctica* and *Thermomyces lanuginosus* lipases. Catalysts.

[27] De Lima L.N., Vieira G.N.A., Kopp W., Tardioli P.W., Giordano R.L.C. (2016). Mono- and heterofunctionalized silica magnetic microparticles (SMMPs) as new carriers for immobilization of lipases. J. Mol. Catal. B Enzym..

[28] Neta N.D.A.S., dos Santos J.C.S., de Oliveira Sancho S., Rodrigues S., Gonçalves L.R.B., Rodrigues L.R., Teixeira J.A. (2012). Enzymatic synthesis of sugar esters and their potential as surface-active stabilizers of coconut milk emulsions. Food Hydrocoll..

[29] Vescovi V., Kopp W., Guisán J.M., Giordano R.L.C., Mendes A.A., Tardioli P.W. (2016). Improved catalytic properties of *Candida antarctica* lipase B multi-attached on tailor-made hydrophobic silica containing octyl and multifunctional amino- glutaraldehyde spacer arms. Process Biochem..

[30] Rahman M., Arumugan M., Khairuddin N.S.K., Abdulmalek E., Basri M., Salleh A. (2012). Microwave assisted enzymatic S-synthesis of fatty acid sugar ester in ionic liquid-tert-butanol biphasic solvent system. Asian J. Chem..

[31] Sutili F.K., Nogueira D.D.O., Leite S.G.F., Miranda L.S.M., De Souza R.O.M.A. (2015). Lipase immobilized in microemulsion based organogels (MBGs) as an efficient catalyst for continuous-flow esterification of protected fructose. RSC Adv..

[32] Siódmiak T., Mangelings D., Vander Heyden Y., Ziegler-Borowska M., Marszałł M.P. (2015). High enantioselective Novozym 435-catalyzed esterification of (R,S)-flurbiprofen monitored with a chiral stationary phase. Appl. Biochem. Biotechnol..

[33] Findrik Z., Megyeri G., Gubicza L., Bélafi-Bakó K., Nemestóthy N., Sudar M. (2016). Lipase catalyzed synthesis of glucose palmitate in ionic liquid. J. Clean. Prod..

[34] Mai N.L., Ahn K., Bae S.W., Shin D.W., Morya V.K., Koo Y.M. (2014). Ionic liquids as novel solvents for the synthesis of sugar fatty acid ester. Biotechnol. J..

[35] Neta N.S., Peres A.M., Teixeira J.A., Rodrigues L.R. (2011). Maximization of fructose esters synthesis by response surface methodology. New Biotechnol..

[36] Pappalardo V.M., Boeriu C.G., Zaccheria F., Ravasio N. (2017). Synthesis and characterization of arabinose-palmitic acid esters by enzymatic esterification. Mol. Catal..

[37] Ortiz C., Ferreira M.L., Barbosa O., dos Santos J.C.S., Rodrigues R.C., Berenguer-Murcia A., Briand L.E., Fernandez-Lafuente R. (2019). Novozym 435: The “perfect” lipase immobilized biocatalyst?. Catal. Sci. Technol..

[38] De Castro H.F., Mendes A.A., dos Santos J.C., de Aguiar C.L. (2004). Modificação de óleos e gorduras por biotransformação. Quim. Nova.

[39] Kumari A., Mahapatra P., Garlapati V.K., Banerjee R. (2009). Enzymatic transesterification of Jatropha oil. Biotechnol. Biofuels.

[40] Fallavena L.P., Antunes F.H.F., Alves J.S., Paludo N., Ayub M.A.Z., Fernandez-Lafuente R., Rodrigues R.C. (2014). Ultrasound technology and molecular sieves improve the thermodynamically controlled esterification of butyric acid mediated by immobilized lipase from *Rhizomucor miehei*. RSC Adv..

[41] Li L., Ji F., Wang J., Li Y., Bao Y. (2015). Esterification degree of fructose laurate exerted by *Candida antarctica* lipase B in organic solvents. Enzym. Microb. Technol..

[42] Kasche V. (1986). Mechanism and yields in enzyme catalysed equilibrium and kinetically controlled synthesis of β-lactam antibiotics, peptides and other condensation products. Enzym. Microb. Technol..

[43] Halling P.J. (1994). Thermodynamic predictions for biocatalysis in nonconventional media: Theory, tests, and recommendations for experimental design and analysis. Enzym. Microb. Technol..

[44] Castillo E., Dossat V., Marty A., Stéphane Condoret J., Combes D. (1997). The role of silica gel in lipase-catalyzed esterification reactions of high-polar substrates. J. Am. Oil Chem. Soc..

[45] Colombié S., Tweddell R.J., Condoret J.S., Marty A. (1998). Water activity control: A way to improve the efficiency of continuous lipase esterification. Biotechnol. Bioeng..

[46] Dossat V., Combes D., Marty A. (1999). Continuous enzymatic transesterification of high oleic sunflower oil in a packed bed reactor: Influence of the glycerol production. Enzym. Microb. Technol..

[47] Marty A., Dossat V., Condoret J.S. (1997). Continuous operation of lipase-catalyzed reactions in nonaqueous solvents: Influence of the production of hydrophilic compounds. Biotechnol. Bioeng..

[48] Séverac E., Galy O., Turon F., Pantel C.A., Condoret J.S., Monsan P., Marty A. (2011). Selection of CalB immobilization method to be used in continuous oil transesterification: Analysis of the economical impact. Enzym. Microb. Technol..

[49] Martins A.B., Schein M.F., Friedrich J.L.R., Fernandez-Lafuente R., Ayub M.A.Z., Rodrigues R.C. (2013). Ultrasound-assisted butyl acetate synthesis catalyzed by Novozym 435: Enhanced activity and operational stability. Ultrason. Sonochem..

[50] Paludo N., Alves J.S., Altmann C., Ayub M.A.Z., Fernandez-Lafuente R., Rodrigues R.C. (2015). The combined use of ultrasound and molecular sieves improves the synthesis of ethyl butyrate catalyzed by immobilized *Thermomyces lanuginosus* lipase. Ultrason. Sonochem..

[51] Martins A.B., Friedrich J.L.R., Cavalheiro J.C., Garcia-Galan C., Barbosa O., Ayub M.A.Z., Fernandez-Lafuente R., Rodrigues R.C. (2013). Improved production of butyl butyrate with lipase from *Thermomyces lanuginosus* immobilized on styrene-divinylbenzene beads. Bioresour. Technol..

[52] Graebin G., Martins B., Garcia-galan C., Fernandez-lafuente R., Ayub M.A.Z., Rodrigues R.C. (2012). Immobilization of lipase B from *Candida antarctica* on porous styrene—divinylbenzene beads improves butyl acetate synthesis. Biotechnol. Prog..

[53] Poppe J.K., Garcia-Galan C., Matte C.R., Fernandez-Lafuente R., Rodrigues R.C., Ayub M.A.Z. (2013). Optimization of synthesis of fatty acid methyl esters catalyzed by lipase B from *Candida antarctica* immobilized on hydrophobic supports. J. Mol. Catal. B Enzym..

[54] Alves J.S., Garcia-Galan C., Schein M.F., Silva A.M., Barbosa O., Ayub M.A.Z., Fernandez-Lafuente R., Rodrigues R.C. (2014). Combined effects of ultrasound and immobilization protocol on butyl acetate synthesis catalyzed by CALB. Molecules.

[55] Tarahomjoo S., Alemzadeh I. (2003). Surfactant production by an enzymatic method. Enzym. Microb. Technol..

[56] Tsukamoto J., Haebel S., Valen G.P., Peter M.G., Franco T.T. (2008). Enzymatic direct synthesis of acrylic acid esters of mono- and disaccharides. J. Chem. Technol. Biotechnol..

[57] Hernandez K., Garcia-Verdugo E., Porcar R., Fernandez-Lafuente R. (2011). Hydrolysis of triacetin catalyzed by immobilized lipases: Effect of the immobilization protocol and experimental conditions on diacetin yield. Enzym. Microb. Technol..

[58] Fernandez-Lorente G., Palomo J.M., Cocca J., Mateo C., Moro P., Terreni M., Fernandez-Lafuente R., Guisan J.M. (2003). Regio-selective deprotection of peracetylated sugars via lipase hydrolysis. Tetrahedron.

[59] Filice M., Bavaro T., Fernandez-Lafuente R., Pregnolato M., Guisan J.M., Palomo J.M., Terreni M. (2009). Chemo-biocatalytic regioselective one-pot synthesis of different deprotected monosaccharides. Catal. Today.

[60] Jönsson L.J., Martín C. (2016). Bioresource technology pretreatment of lignocellulose: Formation of inhibitory by-products and strategies for minimizing their effects. Bioresour. Technol..

[61] Vulfson E.N., Halling P.J., Holland H.L., Rhee J.S., Kwon S.J., Han J.J. (2003). Water activity control for lipase-catalyzed reactions in nonaqueous media. Enzym. Nonaqueous Solvents.

[62] Gandhi N.N., Patil N.S., Sawant S.B., Joshi J.B., Wangikar P.P., Mukesh E.D. (2000). Lipase-catalyzed esterification. Catal. Rev. Sci. Eng..

[63] Mensah P., Carta G. (1999). Adsorptive control of water in esterification with immobilized enzymes. Continuous operation in a periodic counter-current reactor. Biotechnol. Bioeng..

[64] Schmidell W., Lima U.A., Aquarone E., Borzani W. (2001). Biotecnologia Industrial.

[65] Ljunger G., Adlercreutz P., Mattiasson B. (1994). Enzymatic synthesis of octyl-β-glucoside in octanol at controlled water activity. Enzym. Microb. Technol..

[66] Lortie R. (1997). Enzyme catalyzed esterification. Biotechnol. Adv..

[67] Oguntimein G.B., Erdmann H., Schmid R.D. (1993). Lipase catalysed synthesis of sugar ester media. Biotechnol. Lett..

[68] Schlotterbeck A., Lang S., Wray V., Wagner F. (1993). Lipase-catalyzed monoacylation of fructose. Biotechnol. Lett..

[69] Zaidan U.H., Abdul Rahman M.B., Othman S.S., Basri M., Abdulmalek E., Abdul Rahman R.N.Z.R., Salleh A.B. (2012). Biocatalytic production of lactose ester catalysed by mica-based immobilised lipase. Food Chem..

[70] Li L., Ji F., Wang J., Jiang B., Li Y., Bao Y. (2015). Efficient mono-acylation of fructose by lipase-catalyzed esterification in ionic liquid co-solvents. Carbohydr. Res..

[71] Dang H.T., Obiri O., Hayes D.G. (2005). Feed batch addition of saccharide during saccharide-fatty acid esterification catalyzed by immobilized lipase: Time course, water activity, and kinetic model. J. Am. Oil Chem. Soc..

[72] Ward O.P., Fang J., Li Z. (1997). Lipase-catalyzed synthesis of a sugar ester containing arachidonic acid. Enzym. Microb. Technol..

[73] An D., Zhang X., Liang F., Xian M., Feng D., Ye Z. (2019). Synthesis, surface properties of glucosyl esters from renewable materials for use as biosurfactants. Colloids Surf. A.

[74] Rueda N., Dos Santos J.C.S., Torres R., Ortiz C., Barbosa O., Fernandez-Lafuente R. (2015). Improved performance of lipases immobilized on heterofunctional octyl-glyoxyl agarose beads. RSC Adv..

[75] Manoel E.A., dos Santos J.C.S., Freire D.M.G., Rueda N., Fernandez-Lafuente R. (2015). Immobilization of lipases on hydrophobic supports involves the open form of the enzyme. Enzym. Microb. Technol..

[76] Virgen-Ortíz J.J., Tacias-Pascacio V.G., Hirata D.B., Torrestiana-Sanchez B., Rosales-Quintero A., Fernandez-Lafuente R. (2017). Relevance of substrates and products on the desorption of lipases physically adsorbed on hydrophobic supports. Enzym. Microb. Technol..

[77] Martins A.B., Graebin N.G., Lorenzoni A.S.G., Fernandez-Lafuente R., Ayub M.A.Z., Rodrigues R.C. (2011). Rapid and high yields of synthesis of butyl acetate catalyzed by Novozym 435: Reaction optimization by response surface methodology. Process Biochem..

[78] Santos J.C.S.D., Barbosa O., Ortiz C., Berenguer-Murcia A., Rodrigues R.C., Fernandez-Lafuente R. (2015). Importance of the support properties for immobilization or purification of enzymes. ChemCatChem.

[79] Garcia-Galan C., Berenguer-Murcia Á., Fernandez-Lafuente R., Rodrigues R.C. (2011). Potential of different enzyme immobilization strategies to improve enzyme performance. Adv. Synth. Catal..

[80] Liu W.L., Lo S.H., Singco B., Yang C.C., Huang H.Y., Lin C.H. (2013). Novel trypsin–FITC@MOF bioreactor efficiently catalyzes protein digestion. J. Mater. Chem. B.

[81] Idris A., Bukhari A. (2012). Immobilized *Candida antarctica* lipase B: Hydration, stripping off and application in ring opening polyester synthesis. Biotechnol. Adv..

[82] Bilal M., Zhao Y., Noreen S., Shah S.Z.H., Bharagava R.N., Iqbal H.M.N. (2019). Modifying bio-catalytic properties of enzymes for efficient biocatalysis: A review from immobilization strategies viewpoint. Biocatal. Biotransform..

[83] Barbosa O., Ruiz M., Ortiz C., Fernández M., Torres R., Fernandez-Lafuente R. (2012). Modulation of the properties of immobilized CALB by chemical modification with 2,3,4-trinitrobenzenesulfonate or ethylendiamine. Advantages of using adsorbed lipases on hydrophobic supports. Process Biochem..

[84] Rueda N., dos Santos J.C.S., Ortiz C., Torres R., Barbosa O., Rodrigues R.C., Berenguer-Murcia Á., Fernandez-Lafuente R. (2016). Chemical modification in the design of immobilized enzyme biocatalysts: Drawbacks and opportunities. Chem. Rec..

[85] Wagner F.W., Walton M.A.D., Lincoln R.S.M., Lincoln V.H.S. (1991). Separation and Purification of Sugar Esters. U.S. Patent.

[86] Ai M., Xiao N., Jiang A. (2021). Molecular structural modification of duck egg white protein conjugates with monosaccharides for improving emulsifying capacity. Food Hydrocoll..

[87] El-Laithy H.M., Shoukry O., Mahran L.G. (2011). Novel sugar esters proniosomes for transdermal delivery of vinpocetine: Preclinical and clinical studies. Eur. J. Pharm. Biopharm..

[88] Liu K.J. (2017). Enzymatic synthesis of isomaltotriose palmitate and evaluation of its emulsifying property. Enzym. Microb. Technol..

[89] Cavalcanti R.M.F., Silva D.P.D., Paz M.C.D.F., de Queiroz J.C.F. (2017). Screening, production and characterization of biosurfactants from caatinga´s filamentous Fungi. Int. J. Pharm. Sci. Invent..

[90] Beisson F., Tiss A., Rivière C., Verger R. (2000). Methods for lipase detection and assay: A critical review. Eur. J. Lipid Sci. Technol..

[91] Kopp W., Silva F.A., Lima L.N., Masunaga S.H., Tardioli P.W., Giordano R.C., Araújo-Moreira F.M., Giordano R.L.C. (2015). Synthesis and characterization of robust magnetic carriers for bioprocess applications. Mater. Sci. Eng. B Solid-State Mater. Adv. Technol..

[92] Agilent The Essential Chromatography and Spectroscopy Catalog, 2011/2012. https://www.agilent.com/en/promotions/catalog.

[93] Milessi T.S., Perez C.L., Zangirolami T.C., Corradini F.A.S., Sandri J.P., Moreno M.R.F., Giordano R.C., Thevelein J.M., Giordano R.L.C. (2020). Biotechnology for biofuels repeated batches as a strategy for high 2G ethanol production from undetoxified hemicellulose hydrolysate using immobilized cells of recombinant *Saccharomyces cerevisiae* in a fixed—bed reactor. Biotechnol. Biofuels.

