# Supplementary material for: Lipozyme 435-Mediated Synthesis of Xylose Oleate in Methyl Ethyl Ketone
Source: Molecules. 2021 Jun 1;26(11):3317. doi: 10.3390/molecules26113317 (PMC8197991; doi:10.3390/molecules26113317)
Supplement: Supplementary file 1 [file molecules-26-03317-s001.zip › molecules-1229871-supplementary.pdf]

SUPPLEMENTARY MATERIAL

Lipozyme 435-mediated synthesis of xylose oleate in methyl ethyl ketone

Maria Carolina Pereira Gonçalves, Jéssica Cristina Amaral, Roberto Fernandez-Lafuente, Ruy de Sousa Junior, Paulo Waldir Tardioli

Corresponding authors: pwtardioli@ufscar.br (P.W.T.); rfl@icp.csic.es (R.F-L.). Tel.: +55-16-3351-9362 (P.W.T.), +34-915854804 (R.F.-L.)

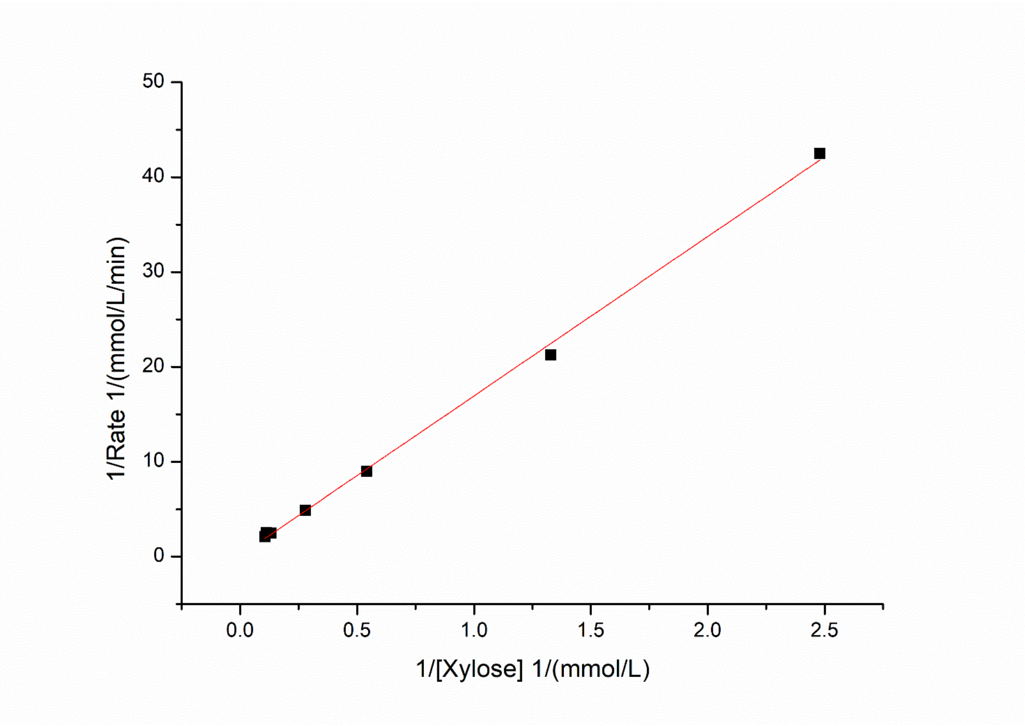

Figure S1. Lineweaver-Burk plot for the initial acylation rates of xylose with oleic acid catalyzed by Lipozyme 435. Reaction conditions: 1.4 mM oleic acid, 60 °C, 200 rpm, 15 min, enzyme load of 0.23% (w/v), with molecular sieves. The rates were calculated as percentage of xylose consumption.

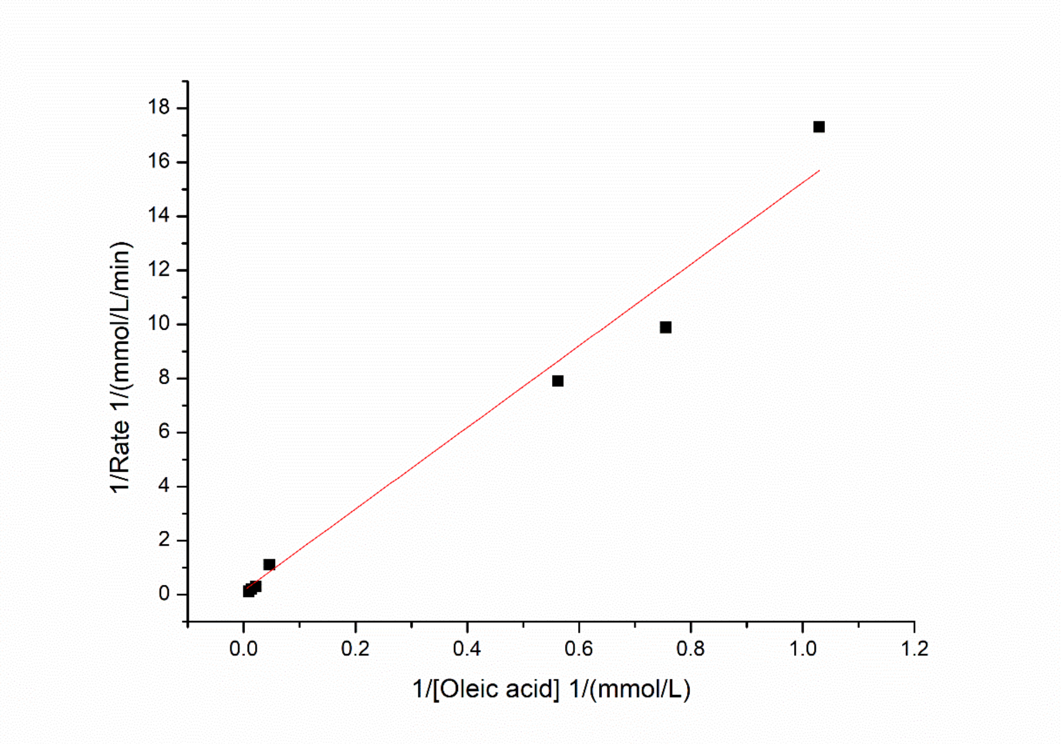

Figure S2. Lineweaver-Burk plot for the initial acylation rates of xylose with oleic acid catalyzed by Lipozyme 435. Reaction conditions: 7 mM xylose, 60 °C, 200 rpm, 15 min, enzyme load of 0.23% (w/v), with molecular sieves. The rates were calculated as percentage of oleic acid consumption.

Table S1. Kinetic constants of the esterification reaction catalyzed by Lipozyme 435. Reaction conditions: 15 min-reaction, 60 °C, 200 rpm, enzyme load of 0.23% (w/v), xylose: oleic acid molar ratio of 1:5, with molecular sieves.

**Parameters**	
V_{max} (mmol/L/min)	6.59 ± 0.21
K_{m} ($_{Xyl}$) (mmol/L)	97.22
K_{m} ($_{OA}$) (mmol/L)	113.24

Xylose monooleate $C_{23}H_{42}O_6$

1:5 $[M-H]^-$ *m/z* 413.2 EC 20V

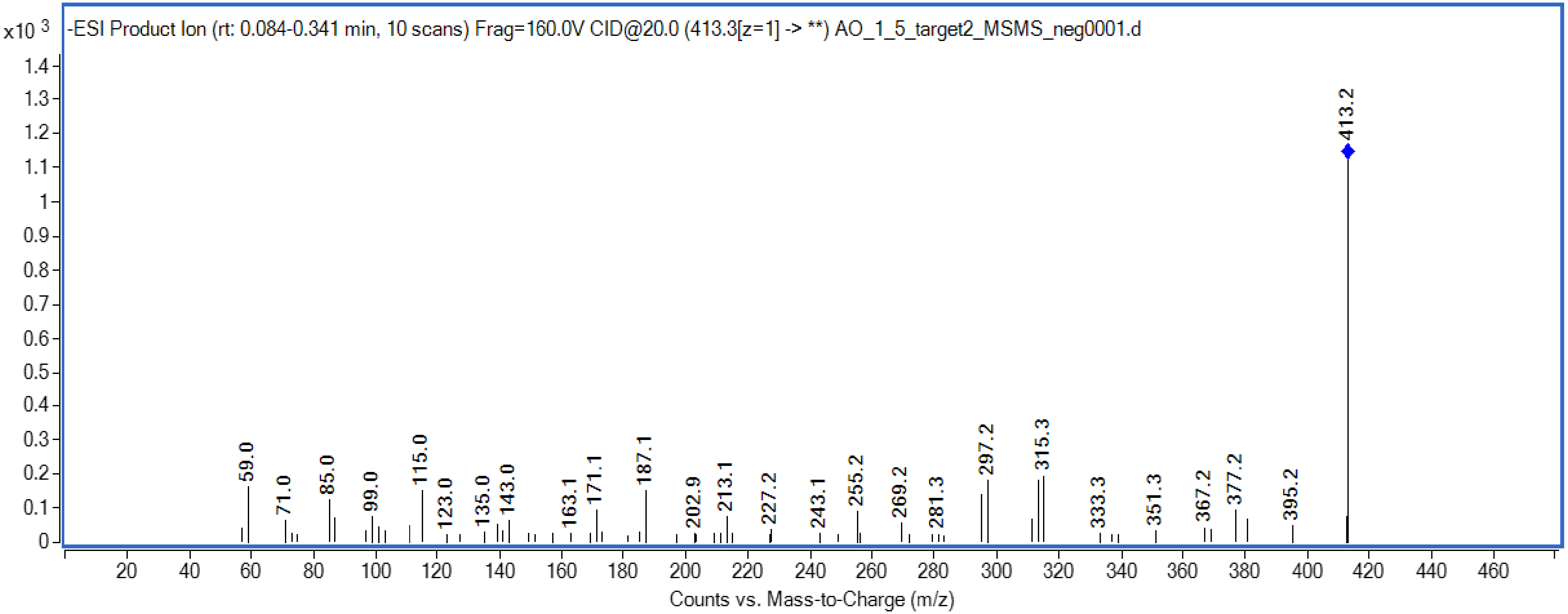

Figure S3. Mass spectrum of xylose monooleate with m/z = 413.2. Xylose: oleic acid molar ratio of 1:5.

Xylose dioleate $C_{41}H_{74}O_7$

1:5 $[M-H]^-$ *m/z* 677.5 EC 22V

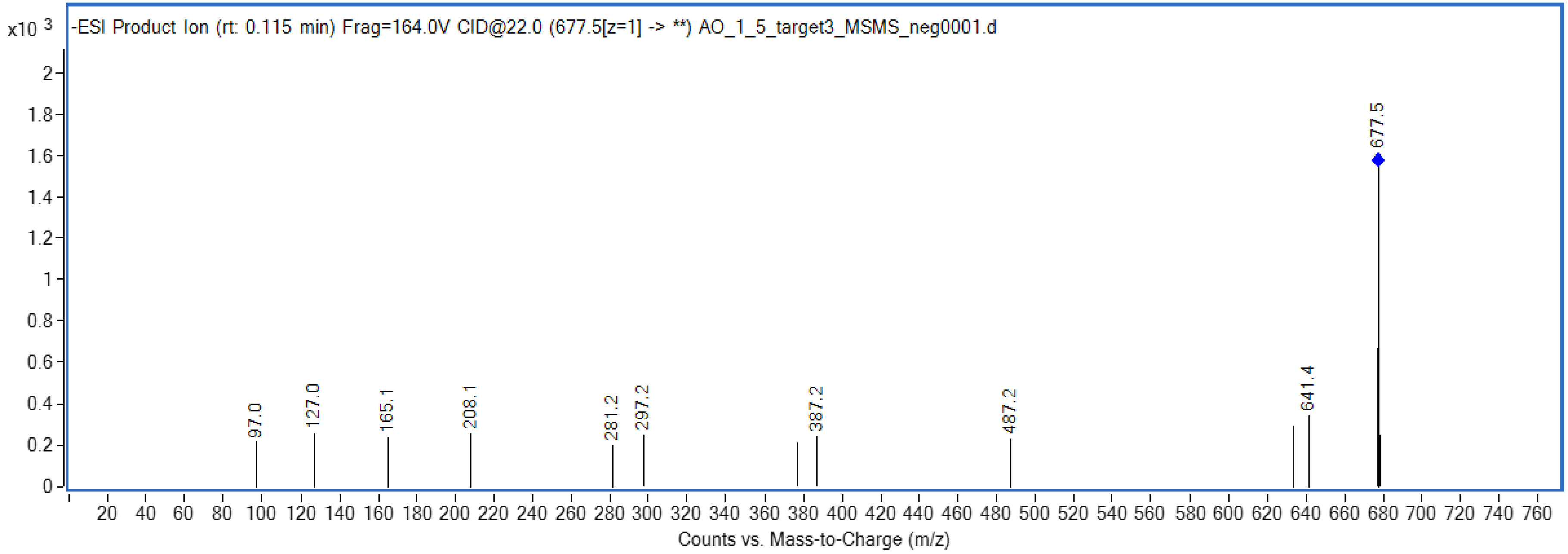

Figure S4. Mass spectrum of xylose dioleate with m/z = 677.5. Xylose: oleic acid molar ratio of 1:5.

Xylose trioleate $C_{59}H_{106}O_8$

1:5 $[M-H]^-$ *m/z* 941.7 EC 20V

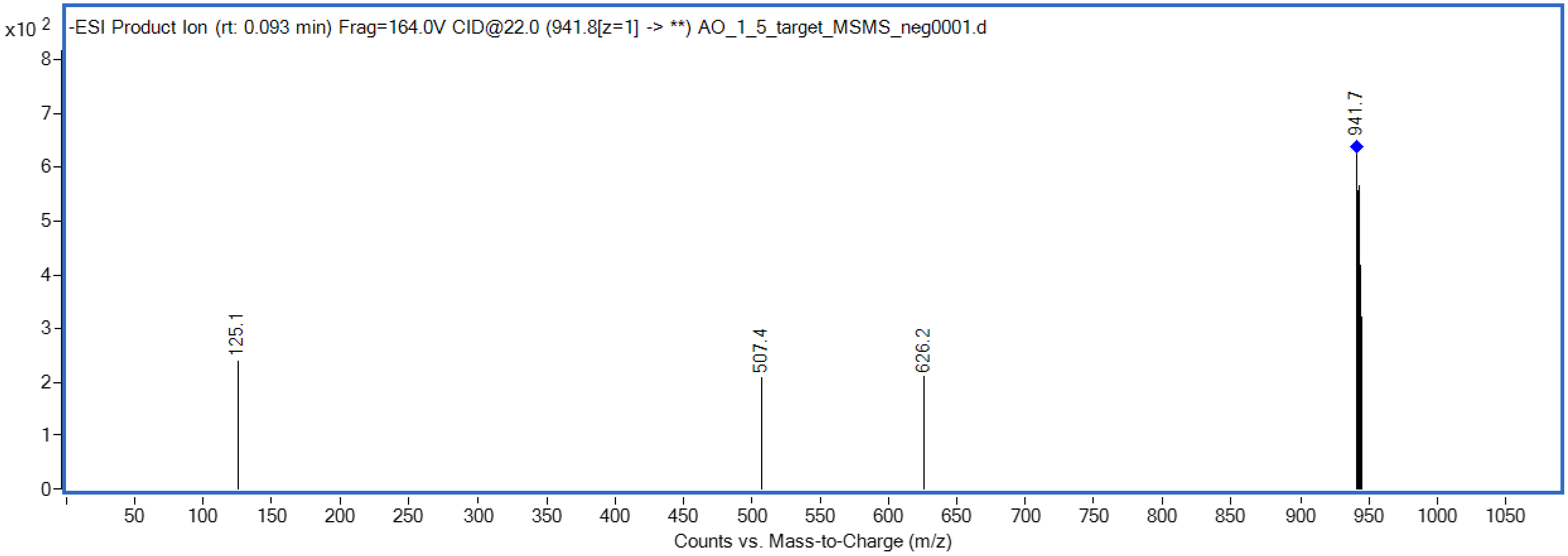

Figure S5. Mass spectrum of xylose trioleate with m/z = 941.7. Xylose: oleic acid molar ratio of 1:5.
